# Filling Teeth

**Published:** 1856-01

**Authors:** F. H. Badger


### ARTICLE II.

### *Filling Teeth.* By F. H. Badger, D. D. S.

In attempting to comply with your request, that I should write out a description of the manner in which I accomplish the filling of cavities in the posterior sides of the second, or third molar teeth, as there is no one better acquainted than yourself, with the necessity of changing the mode to suit the various cases, or the fact, that to write out a description of each, would require a considerable treatise. I have acted upon the supposition that you only wished a description of a single case, that should be one of the most difficult; and, therefore, offer the following, which occurred in my practice in New Orleans. It is also illustrative of other troubles! the hindrances opposed to a satisfactory examination of the teeth, and the consequent liability of decay, in some situations, to escape detection, &c.

On the 4th of March, 1851, Miss H——, of that city, called on me for my attentions to her teeth, which, on several accounts, she was most anxious should embrace every thing I might think it necessary to do to them. Accordingly, in the course of treatment, which required some six weeks, unusual care was taken to discover every defect. Notwithstanding my closest scrutiny, however, the safety of two important teeth would at least have been endangered, but for a disclosure which happened in consequence of extracting the two superior wisdom teeth, which were ruinously decayed in their grinding, and buccal sides.

These two operations it had been supposed would complete the entire series. But upon examining their anterior approximal surfaces after removal, a whitish clouded appearance was observed—in other words, incipient decay was present, at a point where they were previously supposed to be sound. This of course led to a further examination of the posterior sides of their anterior neighbors; or rather a feeling, or probing with an instrument; for nothing more at the time could be done on account of the flow of blood—no cavity was found in either tooth; and I fear that at this time, it would have been something too easy for my patient to have persuaded me that nothing more was necessary, had she been so disposed—not so however, far from it—she was resolutely bent on having me pronounce the teeth certainly *sound*, or to make them so by operation.

The corners of the mouth were alternately stretched back to their utmost capacity, and it was found that it would be impossible to obtain room in this way, even for the eye to rest satisfactorily upon either of the defects, should they really exist.

It was in vain I commented on, enlarged, and as the sequel will prove, exaggerated the difficulties to be encountered in filling, should that operation become necessary, the probable necessity for mutilating the teeth with the file, in order to get at the parts, the extraordinary stretching of the mouth, the suffocating effects of napkins, or other salivary absorbers, her oft-repeated, though unavailing attempts to appease a rebellious disposition to swallow during the operation, &c. All would not do. When I had gotten through with what I intended should be a most terrific expose; the lady smilingly requested to know "when she should call again?" What could I do?

Three weeks afterwards she called, and on passing the mirror behind the teeth, the only available means I had of obtaining a view of the parts, a chalky spot was discovered on each; wonderfully alike in size, shape and position, and both yielding readily to a small cutting instrument.

Now, be it known, these spots, were but slightly removed from the gum, and decidedly nearer the palatine than the buccal surfaces of the teeth. (Dentists will understand this.) They were about the size of a No. 5 shot. The teeth appeared broader on the grinding surface, and narrower at the neck than usual, thus giving their posterior sides an inclination backward, the mouth was small, the saliva abundant.—What was to be done?

I had once in my early practice, Hunter's ideas on the subject being fresh in my memory, extracted a tooth similarly situated, plugged, and returned it to its socket; and although it proved an utter failure, I thought of the plan in the present case; I will not say seriously, but I thought of it, for really I did not know what to do. But being enlisted, there was one thing I was resolved upon, and that was to *do my best.*

In the first place, it was necessary to ascertain whether the decay could with propriety be eradicated with the file. This was "a consummation devoutly to be wished," but one which from appearances there was little room to hope for.

Placing myself nearly in front of my patient, with the mirror in my left hand, and a bent excavator in my right, I went to work on one of the teeth. The spoiled part being comparatively soft, it was not long before sound dentine was reached below; at such a depth, however, as to forbid an operation with the file.

My worst fears were realized—the tooth had to be filled, or lost, perhaps both! I thought of the "fusible metal," and of "metallic paste," and then of "Hill's stopping," as perhaps better than either. But never at that time, having used either of those materials, except the first, by way of experiment, many years since, and then at the instance of an older practitioner, I felt loth to adopt them now, particularly if any thing better could be done.

In the mean time the decay was removed, leaving the cavity a little too much excavated, and ragged at its entrance. To remedy this, a burr-drill was selected, rather larger than the orifice; placed in its handle, and passed through a gold tube, used to cover the shaft of the drill, to prevent friction on the lip, the burr projecting some half an inch beyond its end. Thus prepared, the tube containing the drill, was placed in the corner of the mouth, and forced back until the burr rested on the mouth of the cavity in the tooth; the shaft, forming a little more than a right angle with the row of teeth. The burr was then rotated until about two-thirds imbedded, then removed and the cavity washed out with a syringe, when its entrance was found to be round, smooth, and solid; and a few more touches with an excavator, fitted it for the reception of a plug. By this time I had fixed upon a plan which I intended at least, to *try.*

Abbey's No. 4 gold-foil was folded into a strip, one-third wider than the cavity was deep. The strip was then folded end-wise upon itself until it could be folded no longer, then rolled in the hand with the finger, the proper amount of foil having been used, a pellet was formed the thickness of the burr drill which had been used, its length being the same as the width of the strip out of which it had been formed. It was then forced through a smooth hole in a steel plate, a little too small to allow the burr to pass through, in order to insure its ready entrance into the cavity it was intended to fill, then annealed, and placed conveniently for use.

The necessary preparations having been made to begin, part of a bottle cork was placed between the opposite jaws to prevent the mouth from closing. A pledget of blotting paper was then placed over the mouth of the duct of steno, to prevent, or imbibe the flow of saliva in that quarter, and a small linen napkin also intended to absorb moisture, placed between the lip and gum in front, and carried back between the cheek and jaw, around the tooth to be filled, thence forward over the tongue to the front of the mouth.

With cotton on the end of a carrier for the purpose, and small rolls of tissue paper, carried to the place with a curved tweezers, the cavity was well dried. Having the mirror in the proper place, the pellet was caught with the tweezers, carried back and introduced into the cavity, then pressed home with a flat-ended instrument. A curved instrument was then selected with a tolerably sharp point, with which the pellet was pierced through its centre, to the bottom; other instruments possessing a like curve, but with successively larger points, was forced into the same opening until the gold was brought in close contact with every part of the cavity, and rendered exceedingly dense. The opening in the gold was then filled with a suitable pellet, prepared similar to the first, but rather shorter. This was forced in, a little below the surface of the first pellet which still projected above the mouth of the cavity. The gold overlapping the edges of the orifice, was now gathered up towards the centre, over the outer end of the second pellet and pressed down with a crank shaped instrument with great force.

The napkin, &c., was now removed, and the patient allowed to rest. The gold was then alternately pressed, filed, and burnished, until brought down to a level with the surface of the tooth, and finished.

No moisture was allowed to enter the cavity during the operation.

The plug was hard, and presented as perfect an appearance as any I had ever before inserted. The time occupied in the operation was much less than in some others which had preceded it, where the cavities were much easier of access.

I was delighted, of course, with my success. So was my patient. And two days after the opposite tooth was treated in the same manner.

NASHVILLE, TENN., *Nov. 4th,* 1855.

